# Sleep problems and depression among 237 023 community-dwelling adults in 46 low- and middle-income countries

**DOI:** 10.1038/s41598-019-48334-7

**Published:** 2019-08-19

**Authors:** Andrew Stickley, Mall Leinsalu, Jordan E. DeVylder, Yosuke Inoue, Ai Koyanagi

**Affiliations:** 1 The Stockholm Center for Health and Social Change (SCOHOST), Södertörn University, Huddinge, 141 89 Sweden; 2 Department of Preventive Intervention for Psychiatric Disorders, National Institute of Mental Health, National Center of Neurology and Psychiatry, 4-1-1 Ogawahigashicho, Kodaira, Tokyo 187-8553 Japan; 3 Department of Epidemiology and Biostatistics, National Institute for Health Development, Hiiu 42, 11619 Tallinn, Estonia; 4 Graduate School of Social Service, Fordham University, New York City, NY USA; 5 Carolina Population Center, The University of North Carolina at Chapel Hill, 123 West Franklin St, Chapel Hill, NC 27516 USA; 6 Parc Sanitari Sant Joan de Déu, Universitat de Barcelona, Fundació Sant Joan de Déu, CIBERSAM, Dr Antoni Pujadas, 42, Sant Boi de Llobregat, Barcelona 08830 Spain; 7 ICREA, Pg. Lluis Companys 23, Barcelona, Spain

**Keywords:** Risk factors, Depression

## Abstract

Sleep problems are considered a core symptom of depression. However, there is little information about the comorbidity of sleep problems and depression in low- and middle-income countries (LMICs), and whether sleep problems with depression confer additional risk for decrements in health compared to sleep problems alone. This study thus examined the association between sleep problems and depression and whether sleep problems with depression are associated with an increased risk for poorer health in 46 LMICs. Cross-sectional, community-based data from 237 023 adults aged ≥18 years from the World Health Survey (WHS) 2002–2004 were analyzed. Information on sleep problems (severe/extreme) and International Classification of Diseases 10th Revision depression/depression subtypes was collected. Multivariable logistic (binary and multinomial) and linear regression analyses were performed. Sleep problems were associated with subsyndromal depression (odds ratio [OR]: 2.23, 95% confidence interval [CI]: 1.84–2.70), brief depressive episode (OR = 2.48, 95% CI = 2.09–2.95) and depressive episode (OR = 3.61, 95% CI = 3.24–4.03). Sleep problems with depression (vs. sleep problems alone) conferred additional risk for anxiety, perceived stress and decrements in health in the domains of mobility, self-care, pain, cognition, and interpersonal activities. Clinicians should be aware that the co-occurrence of sleep problems and depression is associated with a variety of adverse health outcomes in LMICs. Detecting this co-occurrence may be important for treatment planning.

## Introduction

Sleep problems, which include difficulty in falling or staying asleep, early morning awakening with the non-resumption of sleep, and an inconsistent sleep/wake pattern^1,2^ are common in the general population. Studies from countries across the world have reported a prevalence of sleep problems ranging from 1.6% to 56.0%^3–5^, while other research has highlighted that sleep problems may be increasing in some countries/populations^6–8^. The fact that many people experience sleep problems is alarming given that they have been linked to a range of detrimental outcomes. Specifically, sleep problems have been associated with major medical conditions such as heart and lung disease, osteoporosis and bodily pain^9^, disability^10^, low quality of life^11^, while there is also evidence that insomnia may result in an increased mortality risk^12,13^.

Sleep problems have also been linked to various mental disorders, including depression^14,15^. Indeed, insomnia is considered a core symptom of depression^16^. Research has indicated that sleep problems/insomnia symptoms such as difficulty initiating and maintaining sleep and early morning awakening are common in mood disorders^17^ and that up to 90% of depressed patients may experience poor quality sleep^18–20^. Importantly, there is also a growing body of evidence that sleep problems may increase the risk for negative outcomes in the context of depression.Studies using data from both clinical and community samples have shown that insomnia/sleep disturbance is linked to a poorer quality of life^21^, functional impairment^22^, as well as an increased risk for suicidal behaviour^23^. There is also some indication that sleep difficulties may affect the course and outcome of depression as they have been linked to the severity of depression^24,25^, poor treatment outcomes (non-remittance)^26,27^, and depression recurrence in older adults^28^.

However, much of the research on the co-existence of sleep problems and depression has been undertaken in Western/high-income countries and there has been comparatively little systematic research on this comorbidity in low- and middle-income countries (LMICs). Two recent community-based studies among adults aged 50 and above in several LMICs found that there was a strong association between sleep problems and depression^5,29^. However, these studies were conducted in a limited number of LMICs and only focused on older adults. In addition, neither study assessed the association between sleep problems and different types of depression, or whether sleep problems comorbid with depression confer additional risk for adverse health outcomes compared to sleep problems alone.

Examining the comorbidity between sleep problems and depression is important in this setting as the prevalence of sleep problems has been reported to be high in many LMICs,^3,5,29^ while it has been estimated that over 80% of the non-fatal disease burden resulting from depression (Years Lived with Disability) occurred in LMICs in 2015^30^. Findings from high-income countries may not be generalizable to LMICs as treatment for both sleep problems and depression is likely to be suboptimal in this setting given that few people receive even minimally adequate treatment for depression alone^31^ and the underlying causes of depression and sleep problems may differ. Indeed, a recent meta-analysis and systematic review has indicated that there is large geographic (continental) variation in the co-occurrence of sleep disturbance and depressive symptoms in older adults with it being much higher in Europe (18.3%) than Africa (4.5%)^32^. The need to understand the association between sleep problems and depression in this setting is given further impetus by the changes being brought by globalization and its accompanying social and economic challenges that might result in increased sleep problems in developing countries^33^. Moreover, as research from the US has indicated that sleep problems in depression are associated with both elevated direct (health care utilization) and indirect costs (e.g. lost work productivity)^34^, determining their association and combined effects in LMICs may have important clinical implications given their potential to place an even greater burden on health services^21^.

The current study thus had two aims: (i) to examine the association between sleep problems and different types of depression in community-dwelling individuals in LMICs; and (ii) to assess whether comorbid sleep problems and depression are associated with greater decrements in a variety of mental and physical health indicators as compared to sleep problems without depression in this setting.

## Data and Methods

### The survey

The World Health Survey (WHS) was a cross-sectional survey undertaken in 2002–2004 in 70 countries. Single-stage random sampling was carried out in 10 countries, while stratified multi-stage random cluster sampling was used in the other 60 countries. Survey details are available from the World Health Organization (WHO) (http://www.who.int/healthinfo/survey/en/). In brief, adults aged ≥18 years with a valid home address were eligible to participate. Kish tables were used to ensure that all household members had an equal chance of being selected. To ensure comparability across countries, the survey questionnaire was subject to standard translation procedures. Face-to-face interviews were conducted by trained interviewers. The individual response rate (ratio of completed interviews among selected respondents after excluding ineligible respondents from the denominator) ranged from 63% (Israel) to 99% (Philippines)^35^. Sampling weights were created using the population distribution as reported by the United Nations Statistical Division to adjust for survey non-response. Ethical boards at each study site provided ethical approval for the survey (see Supplementary Appendix 1) with all participants providing written informed consent. All methods were performed in accordance with relevant ethical guidelines and regulations.

### Primary variables

#### Sleep problems

Sleep problems were assessed by the question “Overall in the last 30 days, how much of a problem did you have with sleeping, such as falling asleep, waking up frequently during the night or waking up too early in the morning?” with answer options none, mild, moderate, severe, and extreme. Those who answered severe and extreme were considered to have sleep problems. This definition has been used in previous publications that have the same survey question on sleep problems^3,5,29^.

### Severity of depressive symptoms

The severity of depressive symptoms was established based on the individual questions of the World Mental Health Survey version of the Composite International Diagnostic Interview (CIDI), which assessed the duration and persistence of depressive symptoms in the past 12 months^36^. Following the algorithms used in a previous WHS publication^37^, four mutually exclusive groups were established based on the ICD-10 Diagnostic Criteria for Research (ICD-10-DCR)^38^ where criterion B referred to symptoms of depressed mood, loss of interest, and fatigability. The algorithms used to define the four groups were the following:

#### Depressive episode group

At least two criterion B symptoms with a total of at least four depressive symptoms lasting two weeks most of the day or all of the day.

#### Brief depressive episode group

Same criteria as depressive episode but did not meet the two-week duration criterion.

#### Subsyndromal depression

At least one criterion B symptom with the total number of symptoms being three or less. A duration criterion of at least two weeks and presence of symptoms during most of the day had to be met.

#### No depressive disorder group

None of the above.

Any depression referred to having subsyndromal depression, brief depressive episode, or depressive episode.

### Health status, perceived stress, anxiety, and observable mental illness

Health status was assessed with the use of 10 health-related questions pertaining to five different domains: (a) mobility; (b) self-care; (c) pain and discomfort; (d) cognition; (e) interpersonal activities. These domains correspond to frequently used health outcome measures included in the Short Form 12 (SF12)^39^, the Health Utilities Index Mark II and III (HUI)^40^ and the EUROQOL 5D^41^, and have been used as indicators of health status in prior studies with these data^42,43^. Each domain consisted of two questions that assessed health function in the past 30 days. Each item was scored on a five-point scale ranging from ‘none’ to ‘extreme/cannot do’. In accordance with previous WHS publications^44,45^, we assessed perceived stress in the last month with the use of two questions which were taken from the Perceived Stress Scale^46^. Each question was scored on a five-point scale which ranged from ‘never’ to ‘very often’. The actual questions used to assess health status and perceived stress can be found in Supplementary Appendix 2. For perceived stress and each separate domain for health status, we used factor analysis with polychoric correlations to obtain a factor score which was later converted to scores ranging from 0–100^45^ with higher values representing higher levels of perceived stress or worse health function. Anxiety was assessed by the question ‘Overall in the past 30 days, how much of a problem did you have with worry or anxiety’ with response options: none, mild, moderate, severe, and extreme. In accordance with previous WHS publications, those who answered severe and extreme were considered to have anxiety^3,47^. Observable mental illness was based on the interviewer’s subjective impression of the presence of mental health problems at the conclusion of the interview.

### Control variables

The control variables used in the analysis were selected based on past literature and included sex, age, education, wealth, residential location (rural or urban), physical activity, alcohol consumption, current smoking (no, non-daily, daily), obesity, and chronic physical conditions^48,49^. Education was based on the highest level of education attained (no formal education, primary education, secondary or high school completed, and tertiary education completed). Country-wise wealth quintiles were created using principal component analysis based on 15–20 assets depending on the country. Physical activity was assessed with the International Physical Activity Questionnaire, and was categorized as high, moderate, and low based on conventional cut-offs. Alcohol consumption was first assessed by the screening question ‘Have you ever consumed a drink that contains alcohol (such as beer, wine, etc.)?’ Respondents who replied negatively were considered lifetime abstainers. If the respondent replied affirmatively, then he/she was asked how many standard drinks of any alcoholic beverage he/she had on each day of the past seven days. The number of days in the past week on which four (female) or five (male) drinks were consumed was calculated, and a total of 1–2 days and three days or more in the past seven days were considered infrequent and frequent heavy drinking, respectively^3^. With the exception of lifetime abstainers, all other respondents were considered to be non-heavy drinkers. Obesity was defined as a body mass index of ≥30 kg/m^2^ based on self-reported weight and height. Seven chronic physical conditions (angina, arthritis, asthma, chronic back pain, diabetes, visual impairment, hearing problems) were assessed and those having at least one of the conditions were considered to have a chronic condition. Arthritis, asthma, and diabetes were based solely on self-reported lifetime diagnosis. For angina, in addition to a self-reported diagnosis, a symptom-based diagnosis based on the Rose questionnaire was also used^50^. Chronic back pain was defined as back pain (including disc problems) every day during the last 30 days. Visual impairment was defined as extreme difficulty in seeing and recognizing a person that the participant knows across the road (i.e., from a distance of about 20 meters)^51^. Hearing problems were considered to be present if the interviewer observed this condition at the conclusion of the survey.

### Statistical analysis

From the 69 countries for which data were publicly available, 10 were excluded due to an absence of sampling information. Furthermore, 8 high-income countries were omitted as the short version of the questionnaire was administered in these countries and they lacked data on the majority of the variables used in this study. Spain and the United Arab Emirates were also omitted to focus on LMICs. Finally, Turkey, Latvia, and Morocco were excluded as data on some of the control variables were not collected. Thus, the final sample consisted of 237 023 individuals from 46 LMICs. Based on the World Bank classification at the time of the survey, this corresponded to 21 low-income countries (n = 105 286) and 25 middle-income countries (n = 131 737). The included countries and their sample size are shown in Supplementary Appendix 3. With the exception of China, Comoros, Congo, Ivory Coast, India, and Russia, these data are nationally representative.

Statistical analyses were performed with Stata 14.1 (Stata Corp LP, College station, Texas). Multivariable multinomial logistic regression analysis using the overall sample was conducted to assess the association between sleep problems (exposure) and the type of depression (outcome). Three models were constructed to assess the influence of the inclusion of different variables into the model on the sleep problem-depression relationship: Model 1 - adjusted for sociodemographic factors (age, sex, education, wealth, residential location, and country); Model 2 - adjusted for the variables in Model 1 and health behaviour (physical activity, alcohol consumption, smoking); Model 3 - adjusted for the variables in Model 2, obesity, and chronic physical conditions.

Next, in order to assess whether the association between sleep problems and any depression is consistent across countries, we conducted country-wise binary logistic regression analysis adjusting for age and sex. The estimates for each country were also combined into a random-effect meta-analysis with the Higgins’s *I*^2^ statistic being calculated. Higgins’s *I*^2^ represents the degree of heterogeneity between countries that is not explained by sampling error with a value of 25% often considered as low, 50% and above as moderate and over 75% as indicating high heterogeneity^52^.

Finally, we conducted separate multivariable linear regression analyses where each of the five health function domains and perceived stress were outcomes and a three-category variable based on a combination of sleep problems and any depression [(a) no sleep problems and no depression; (b) any sleep problems without depression; (c) any sleep problems with depression] was the exposure variable, as well as additional binary logistic regression analyses using observable mental illness and anxiety as the outcome. We used sleep problems without depression as the reference category as our main aim was to assess whether sleep problems with depression confers an additional risk for various health outcomes when compared with sleep problems in the absence of depression. We did not include those with depression without sleep problems in this part of the analysis for this same reason. Adjustment for age, sex, education, wealth, residential location, physical activity, alcohol consumption, smoking, obesity, chronic physical conditions, and country was done. Brazil, Hungary, and Zimbabwe were not included in the analysis with perceived stress as the outcome due to a lack of data.

Adjustment for country was conducted by including dummy variables for each country^45^. To avoid the omission of a large number of individuals from the regression analyses, a missing category was included only for obesity as 29.8% of the data was missing. Taylor linearization methods were used in all analyses to account for the sample weighting and complex study design. Results from linear and logistic regression analyses are presented as β-coefficients and odds ratios (ORs) respectively, with 95% confidence intervals (CIs). The level of statistical significance was *p* < 0.05.

## Results

**Figure 1:**
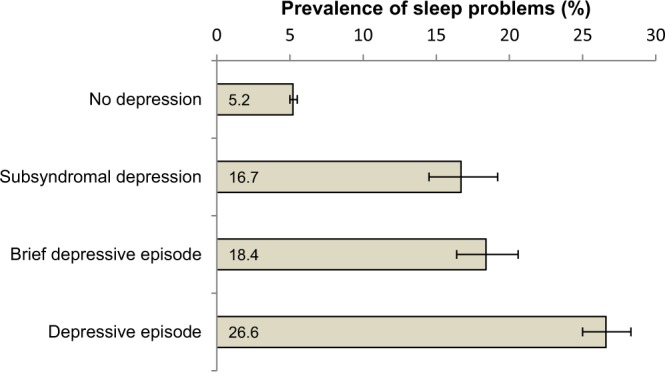
Prevalence of sleep problems by different types of depression. Estimates are based on weighted sample. Bars denote 95% confidence interval.

The mean (SD) age of the participants was 38.4 (16.0) years; 50.8% were female. The sample characteristics are presented in Table 1. The prevalence of sleep problems and any depression was 7.5% and 11.7%, respectively. Sleep problems were much more common among those with all types of depression with a particularly high prevalence among those with a depressive episode [26.6% vs. 5.2% (no depression)] (Fig. 1). The multivariable analysis showed that sleep problems were significantly associated with all types of depression even after adjustment for sociodemographic factors, health behaviour, obesity, and chronic physical conditions (Model 3) (Table 2). Specifically, the ORs (95% CI) for subsyndromal depression, brief depressive episode, and depressive episode were 2.23 (1.84–2.70), 2.48 (2.09–2.95), and 3.61 (3.24–4.03), respectively (Model 3).

**Table 1:** Sample characteristics.

Characteristic	Category	
Sleep problems	Yes	7.5
Depression type	Subsyndromal depression	2.5
	Brief depressive episode	2.7
	Depressive episode	6.5
Age (years)	Mean (SD)	38.4 (16.0)
Sex	Female	50.8
Education	No formal	26.1
	Primary	31.0
	Secondary	33.7
	Tertiary	9.2
Wealth	Poorest	20.1
	Poorer	20.0
	Middle	19.9
	Richer	20.0
	Richest	20.0
Residential location	Urban	43.1
Physical activity	High	63.2
	Moderate	19.4
	Low	17.4
Alcohol consumption	Lifetime abstainer	66.1
	Non-heavy	29.1
	Infrequent heavy	3.8
	Frequent heavy	1.1
Current smoking	Non-daily	5.8
	Daily	20.9
Obesity	Yes	9.0
Chronic physical condition	Yes	31.6

Abbreviation: SD Standard deviation.

Data are % unless otherwise stated.

Estimates are based on weighted sample.

**Table 2:** Association between sleep problems and different types of depression (outcome) estimated by multivariable multinomial logistic regression.

	Depression subtypes (Reference = No depression)					
	Subsyndromal depression		Brief depressive episode		Depressive episode	
	OR	[95% CI]	OR	[95% CI]	OR	[95% CI]
Model 1	2.64*	[2.21, 3.15]	2.85*	[2.43, 3.34]	4.48*	[4.04, 4.96]
Model 2	2.58*	[2.13, 3.12]	2.87*	[2.42, 3.40]	4.39*	[3.93, 4.89]
Model 3	2.23*	[1.84, 2.70]	2.48*	[2.09, 2.95]	3.61*	[3.24, 4.03]

Abbreviation: OR Odds ratio; CI Confidence interval.

Model 1: Adjusted for age, sex, education, wealth, setting, and country.

Model 2: Adjusted for the factors in Model 1 and physical activity, alcohol consumption, and smoking.

Model 3: Adjusted for the factors in Model 2, obesity, and chronic physical conditions.

*p < 0.001.

**Figure 2:**
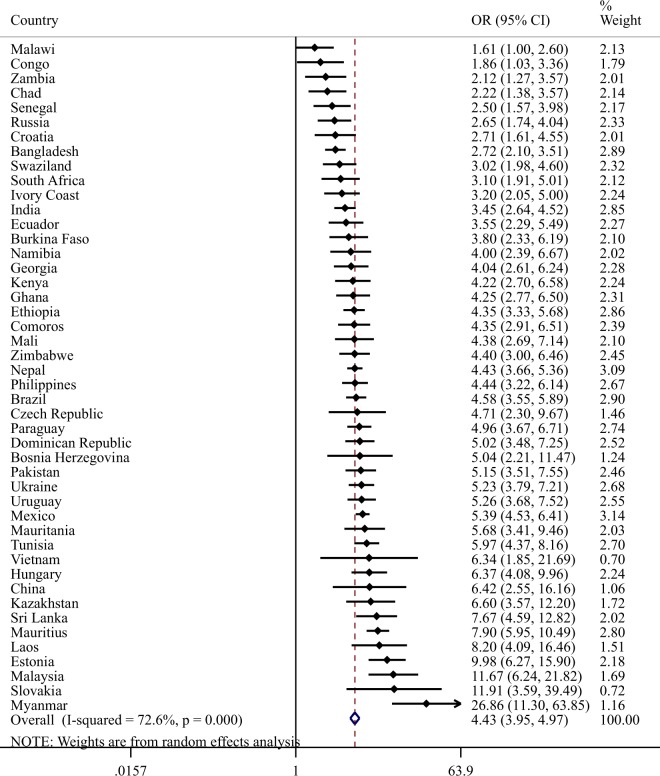
Association between sleep problems and depression estimated by multivariable binary logistic regression adjusting for age and sex. Abbreviation: OR Odds ratio; CI Confidence interval. The pooled estimate was calculated by meta-analysis with random effects. Depression referred to having subsyndromal depression, brief depressive episode, or depressive episode.

Country-wise analyses showed that sleep problems were significantly associated with any depression in all countries except Malawi. A moderately-high level of between-country heterogeneity was observed (I^2^ = 72.6%) with the pooled estimate based on a meta-analysis being OR = 4.43 (95% CI = 3.95–4.97) (Fig. 2). The association between different combinations of sleep problems and depression with a variety of health outcomes estimated by multivariable regression analyses is shown in Table 3. Sleep problems with depression was associated with significant decrements in all 5 health outcomes and higher levels of stress, observable mental illness and anxiety compared to sleep problems without depression.

**Table 3:** Association of different combinations of sleep problems and depression with various health outcomes.

Outcome	No sleep problems or depression	Sleep problems without depression	Sleep problems with depression
Binary logistic regression analysis	OR [95% CI]		OR [95% CI]
Observable mental illness	0.40* [0.30, 0.52]	Ref.	2.09* [1.50, 2.92]
Anxiety	0.13* [0.12, 0.15]	Ref.	3.27* [2.70, 3.96]
Linear regression analysis^a^	β [95% CI]		β [95% CI]
Mobility	−18.21* [−19.49, −16.94]	Ref.	8.26* [6.18, 10.33]
Self-care	−14.77* [−16.14, −13.39]	Ref.	9.10* [6.55, 11.66]
Pain and discomfort	−20.68* [−21.92, −19.44]	Ref.	7.56* [5.60, 9.51]
Cognition	−18.19* [−19.62,−16.76]	Ref.	10.20* [7.74,12.66]
Interpersonal activities	−14.01* [−15.49, −12.54]	Ref.	7.92* [5.25,10.60]
Perceived stress^b^	−8.97* [−10.25, −7.69]	Ref.	10.00* [7.93, 12.07]

Abbreviation: OR Odds ratio; CI Confidence interval; Ref. Reference category.

Depression referred to having subsyndromal depression, brief depressive episode, or depressive episode.

All models are adjusted for age, sex, education, wealth, residential location, physical activity, alcohol consumption, smoking, obesity, chronic physical conditions, and country.

^a^All outcomes ranged from 0–100 with higher scores indicating worse conditions.

^b^Brazil, Hungary, and Zimbabwe are not included due to lack of data on perceived stress.

## Discussion

This study showed that compared to individuals without sleeping difficulties, the prevalence of depression was significantly elevated in people with sleep problems. The odds for having subsyndromal depression among people with sleep problems more than doubled, and this odds ratio was 3.6 times higher for depressive episode. Sleep problems were associated with significantly increased odds for any depression in 45 of the 46 countries included in the study. Sleep problems with depression (vs. sleep problems alone) conferred additional risk for observable mental illness, anxiety, perceived stress and decrements in health status in the domains of mobility, self-care, pain, cognition, and interpersonal activities.

The finding that sleep problems were associated with significantly increased odds for depression concurs with earlier Western population-based studies which have linked sleep problems and depression/mood disorders in cross-sectional analyses^53,54^ It also accords with the results from previous cross-sectional studies undertaken in several LMICs among young^33^ and older adults^5,29^ which found that there was a strong association between sleep problems and depression, but that the strength of the association (as measured by the odds ratio) varied across countries^5,29^. The exact way in which poorer sleep and depression are linked is uncertain. Although insomnia/hypersomnia is classified as one of the symptoms of major depressive disorder (MDD) in DSM-5^16^, findings from a number of recent prospective studies have indicated that insomnia may also be a risk factor for mental illness, including depression, in its own right^14,55,56^ and that the relationship between depression and insomnia might therefore be bidirectional^57,58^. A detailed discussion of the mechanisms underlying this association is beyond the scope of this paper although genetic^59^ and biological factors (e.g. dysregulation of the hypothalamic-pituitary-adrenal (HPA) axis)^60^ have both been proposed as being important in the association between sleep problems and depression. In addition, it has been suggested that problems in regulating emotions might also play a role in linking sleep problems and depression^61^.

As yet, the research on whether sleep problems and comorbid depression might have an especially detrimental impact on health and well-being has been somewhat limited. Thus, the finding that those with sleep problems and co-occurring depression had a significantly increased risk for a variety of negative health outcomes including worse cognition, mobility, greater pain and more perceived stress compared to individuals both without sleep problems or depression *and* with sleep problems but without depression has significant public health implications. Importantly, this result accords with those from several earlier studies which showed that individuals with comorbid sleep disorders/insomnia and depressive/mood disorders were more likely to have a lower quality of life^62,63^ and greater impairment in several (but not all) functional domains compared to those with no or less severe sleep problems^22,64^. It can only be speculated what underlies this finding. Previous research has indicated that sleep problems are associated with more severe depressive symptoms^25^ and possibly treatment resistance in depression^65^ both of which might be important for detrimental health outcomes. In addition, as both sleep problems^15,66^ and depression^67^ are independently associated with worse outcomes such as impaired role/social functioning, it is possible that when comorbid, their detrimental effects might be additive or synergistic.

This study has several strengths. It used data from mostly representative population samples from a large number of countries, which were collected using a common research methodology and instruments. In addition, it was also possible to examine a variety of outcomes while controlling for a number of covariates. Indeed, to the best of our knowledge it is the first study to examine the association between sleep problems and different forms of depression and how they are associated with various health outcomes in multiple LMICs. It should be acknowledged however, that there are also a number of study limitations. For practical considerations, information on sleep was not collected using polysomnography or actigraphy^68^ but was rather obtained from self-reports. However, there is evidence that depressed individuals may report some aspects of their sleep quality/problems inaccurately^69,70^. We also used a single-item question to collect information on sleep. Although this measure related to different sleep problems (difficulty falling asleep, waking at night and early morning awakening), we were not able to examine the effects of these sleep problems individually despite the fact that the strength of the depression-insomnia association may differ across different sleep problems^57^. It is also possible that variables were missing from the analysis that might have been influential in the observed associations. For example, childhood adversities have been linked to an increased risk for both poor quality sleep^71^ and depression^72^ in adulthood. Finally, as this study was cross-sectional it was not possible to establish causality or determine the order in which the observed associations occurred. Prospective research is now needed in LMICs to further elucidate the association between sleep problems and depression both in clinical and community-based samples and how they impact on health and well-being in these countries.

The finding that there is a strong association between sleep problems and depression in LMICs and that the comorbidity of these conditions is linked to an increased risk for a number of negative health outcomes highlights the importance of detecting and treating insomnia in depressed individuals in LMICs, especially as there is some evidence that interventions to improve sleep may have a rapid effect on depression^73^. In terms of this, a combined therapeutical approach is generally suggested for these comorbid conditions^74^ with research indicating that interventions such as cognitive-behavioural therapy for insomnia (CBT-I) and pharmacological treatments may be beneficial for depressed patients with sleep problems^75–77^. However, it should be recognized that the widespread adoption of such an approach may prove difficult given the many challenges involved in effectively treating mental illness and its comorbidities in resource-limited countries^78^. Given this, and other factors, such as the stigma attached to mental ill health^78^ that can prevent help-seeking behaviour, the fact that insomnia may be a prospective risk factor for depression and other negative health outcomes^79^, might indicate that early interventions for sleep problems may also be efficacious for mental health^80^ in LMICs.

## Supplementary information

Supplementary information

## Data Availability

The WHS is a dataset that is publicly available upon request.
