# Supplementary material for: Sleep problems and depression among 237 023 community-dwelling adults in 46 low- and middle-income countries
Source: Sci Rep. 2019 Aug 19;9:12011. doi: 10.1038/s41598-019-48334-7 (PMC6700183; doi:10.1038/s41598-019-48334-7)
Supplement: Supplementary file 1 — Supplementary information [file 41598_2019_48334_MOESM1_ESM.pdf]

Supplementary information

Sleep problems and depression among 237 023 community-dwelling adults in 46 low- and middle-income countries

Andrew Stickley, Mall Leinsalu, Jordan E. DeVylder, Yosuke Inoue, Ai Koyanagi

Appendix 1 Ethical Approval Committees for the World Health Survey

Ethical approval was obtained from each of the following committees in the respective countries for the World Health Survey:

Country	Ethical Approval Committee
Bangladesh	Mitra and Associates
Bosnia Herzegovina	The Federal Public Health Institute
Brazil	Fundacao Oswaldo Cruz
Burkina Faso	Institut de Recherche en Sciences de la Santé
Chad	Faculté des Sciences de la Santé, Univ N'Djamena
China	Centre for Health Statistics Information
Comoros	Bureau Comorien de Conseil
Croatia	The Croatian National Institute of Public Health
Czech Republic	Institute of Health Information and Statistics
Dominican Republic	Centro de Estudios Sociales y Demográficos (CESDEM)
Ecuador	Fundación Ecuatoriana para la Salud y el Desarroll (FESALUD)
Estonia	Saar Poll Ltd
Ethiopia	Department of Community Health, Jimma University
Georgia	Georgian State Medical Academy (GSMA)
Ghana	Department of Community Health, Ghana Medical School
Hungary	Johan Bela National Centre for Epidemiology
India	International Institute of Population Sciences
Ivory Coast	Ministère de la Santé
Kazakhstan	Kazakstan School of Public Health (KSPH)
Kenya	Central Bureau of Statistics
Laos	National Institute of Public Health, Ministry of Health
Malawi	Centre for Social Research (CSR)
Malaysia	Public Health Institute, Ministry of Health
Mali	Cellule de Planification et de Statistique, (CPS)
Mauritania	Office Nationale de la Statistique (ONS)
Mauritius	Mauritius Institute of Health
Mexico	Instituto Nacional de Salud Pública
Myanmar	Department of Medical Research, Ministry of Health
Namibia	Ministry of Health
Nepal	ORG -MARG Nepal PVT. Ltd
Pakistan	Ministry of Health
Paraguay	Fac.de Ciencias Veterinarias, Univ. Nacional/DGEEC
Philippines	College of Medicine, University of the Philippines
Republic of Congo	Unité de recherche sur les systèmes de santé
Russia	Semashko Institute for Research on Social Hygiene
Senegal	Direction Etudes, Recherche et Formation (DERF)
Slovakia	Environment, s. c., Centre of Biostatistics and Envi
South Africa	Community Agency for Social Enquiry (CASE)
Sri Lanka	Ministry of Health

Swaziland	Faculty of Health Sciences, Univ Swaziland
Tunisia	Institut National de la Santé Publique
Ukraine	Odessa State Medical University
Uruguay	Centro de Estudios de Economia y Salud (CEES)
Vietnam	Ministry of Health
Zambia	School of Humanities & Social Sciences, University of Zambia
Zimbabwe	Community Health, University of Zimbabwe

Appendix 2 Questions used to assess health status and perceived stress

**Mobility**	(1) Overall in the last 30 days, how much difficulty did you have with moving around?<br>(2) In the last 30 days, how much difficulty did you have in vigorous activities, such as running 3 km (or equivalent) or cycling?
**Self-care**	(1) Overall in the last 30 days, how much difficulty did you have with self-care, such as washing or dressing yourself?<br>(2) In the last 30 days, how much difficulty did you have in taking care of and maintaining your general appearance (e.g. grooming, looking neat and tidy etc.)
**Pain and discomfort**	(1) Overall in the last 30 days, how much of bodily aches or pains did you have?<br>(2) In the last 30 days, how much bodily discomfort did you have?
**Cognition**	(1) Overall in the last 30 days, how much difficulty did you have with concentrating or remembering things?<br>(2) In the last 30 days, how much difficulty did you have in learning a new task (for example, learning how to get to a new place, learning a new game, learning a new recipe etc.)?
**Interpersonal activities**	(1) Overall in the last 30 days, how much difficulty did you have with personal relationships or participation in the community?<br>(2) In the last 30 days, how much difficulty did you have in dealing with conflicts and tensions with others?
**Perceived stress**	(1) In the last month, how often have you felt that you were unable to control the important things in your life?<br>(2) In the last month, how often have you found that you could not cope with all the things that you had to do?

Appendix 3 Countries included in the study and their sample size

Country	N	Country	N
Bangladesh	5,942	Mali	4,886
Bosnia and Herzegovina	1,031	Mauritania	3,902
Brazil	5,000	Mauritius	3,968
Burkina Faso	4,948	Mexico	38,746
Chad	4,870	Myanmar	6,045
China	3,994	Namibia	4,379
Comoros	1,836	Nepal	8,820
Croatia	993	Pakistan	6,501
Czech Republic	949	Paraguay	5,288
Dominican Republic	5,027	Philippines	10,083
Ecuador	5,675	Republic of Congo	3,075
Estonia	1,020	Russia	4,427
Ethiopia	5,089	Senegal	3,461
Georgia	2,950	Slovakia	2,535
Ghana	4,165	South Africa	2,629
Hungary	1,419	Sri Lanka	6,805
India	10,687	Swaziland	3,117
Ivory Coast	3,251	Tunisia	5,202
Kazakhstan	4,499	Ukraine	2,860
Kenya	4,640	Uruguay	2,996
Laos	4,988	Vietnam	4,174
Malawi	5,551	Zambia	4,165
Malaysia	6,145	Zimbabwe	4,290
